# Preventive and Therapeutic Autoantibodies Protect against Neuronal Excitotoxicity

**DOI:** 10.20900/jpbs.20230006

**Published:** 2023-06-25

**Authors:** Xianjin Zhou

**Affiliations:** Department of Psychiatry, University of California San Diego, La Jolla, CA 92093, USA;

**Keywords:** anti-NMDAR1 autoantibody, antibody IgM isotype, excitotoxicity, early prevention, neurological disease, psychiatric disorder

## Abstract

High titers of anti-NMDAR1 IgG autoantibodies were found in the brains of patients with anti-NMDAR1 encephalitis that exhibits psychosis, impaired memory, and many other psychiatric symptoms in addition to neurological symptoms. Low titers of blood circulating anti-NMDAR1 IgG autoantibodies are sufficient to robustly impair spatial working memory in mice with intact blood-brain barriers (BBB). On the other hand, anti-NMDAR1 autoantibodies were reported to protect against neuronal excitotoxicity caused by excessive glutamate in neurological diseases. Activation of extrasynaptic NMDARs is responsible for neuronal excitotoxicity, whereas activation of synaptic NMDARs within the synaptic cleft is pro-survival and essential for NMDAR-mediated neurotransmission. Unlike small IgG, IgM antibodies are large and pentameric (diameter of ~30 nm). It is plausible that IgM anti-NMDAR1 autoantibodies may be restricted to bind extrasynaptic NMDARs and thereby specifically inhibit neuronal excitotoxicity, but physically too large to enter the synaptic cleft (width: 20–30 nm) to suppress synaptic NMDAR-mediated neurotransmission in modulation of cognitive function and neuronal pro-survival signaling. Hence, blood circulating anti-NMDAR1 IgM autoantibodies are both neuroprotective and pro-cognitive, whereas blood circulating anti-NMDAR1 IgG and IgA autoantibodies are detrimental to cognitive function. Investigation of anti-NMDAR1 IgM autoantibodies may open up a new avenue for the development of long-lasting preventive and therapeutic IgM anti-NMDAR1 autoantibodies that protect from neuronal excitotoxicity in many neurological diseases and psychiatric disorders.

## INTRODUCTION

High titers of anti-NMDAR1 IgG autoantibodies are found in the brains of patients with anti-NMDAR1 encephalitis that exhibits psychosis, impaired memory, and many other psychiatric symptoms in addition to neurological symptoms [1]. To investigate potential effects of blood J circulating anti-NMDAR1 autoantibodies on cognitive function and behaviors, we generated mice carrying low titers of anti-NMDAR1 autoantibodies, predominantly IgG isotype, in blood against a single P2 antigenic epitope of mouse NMDAR1 using active immunization [2]. Mice carrying the anti-NMDAR1 autoantibodies are healthy and display no differences in locomotion, sensorimotor gating, contextual memory, and depression-like phenotypes compared to controls. Presence of low titers of blood circulating anti-NMDAR1 autoantibodies, however, is sufficient to robustly impair spatial working memory (*p* = 2.02 × 10^−8^, power: 1) with a large effect size (*d* = 2.3) in mice with intact BBB, suggesting that the BBB may be insufficient to block crossover of blood circulating anti-NMDAR1 autoantibodies to brain parenchyma. Consistently, ~0.1%–0.2% of blood circulating antibodies were reported to nonspecifically cross the BBB regardless of antibody IgG or IgM isotype [3–5]. Interestingly, low titers of natural anti-NMDAR1 autoantibodies were reported in the blood of ~5–10% of the general human population and psychiatric patients [6–9]. However, these natural anti-NMDAR1 autoantibodies are reported to be predominantly IgM or IgA isotype. Since antibodies persist for months and years in human blood, whether chronic presence of these blood circulating natural anti-NMDAR1 autoantibodies may impact human cognitive function remains to be investigated [10].

## EXCITOTOXICITY

Neuronal excitotoxicity is the primary mechanism for neuronal injury in neurological diseases [11] (e.g., stroke, epilepsy) and neurodegenerative diseases [12] (e.g., AD, PD, HD, ALS), and also contribute to the pathogenesis of psychiatric disorders such as schizophrenia [13,14], PTSD [15], etc. Activation of extrasynaptic NMDARs outside of the synapses is responsible for neuronal excitotoxicity, whereas activation of synaptic NMDARs within the synaptic cleft is responsible for neurotransmission and promotes neuronal survival [16,17]. Memantine, an NMDAR antagonist that preferentially (but not specifically) inhibits extrasynaptic NMDARs [18], has been used to suppress neuronal excitotoxicity in Alzheimer’s disease. Suppressing neuronal excitotoxicity with a low dose of memantine improves cognitive functions in healthy persons [19,20], schizophrenia patients [21–23], and animal models including mice [24,25], rats [20,26], and chicks [27]. These studies indicated that inhibition of extrasynaptic NMDARs not only suppresses neuronal excitoxicity in diseases and but also enhances cognitive function in healthy humans and animals. None of small molecule NMDAR antagonists, however, is capable of specifically inhibiting extrasynaptic NMDARs without suppressing synaptic NMDARs.

## ANTI-NMDAR1 AUTOANTIBODY ISOTYPES

**Figure 1.**
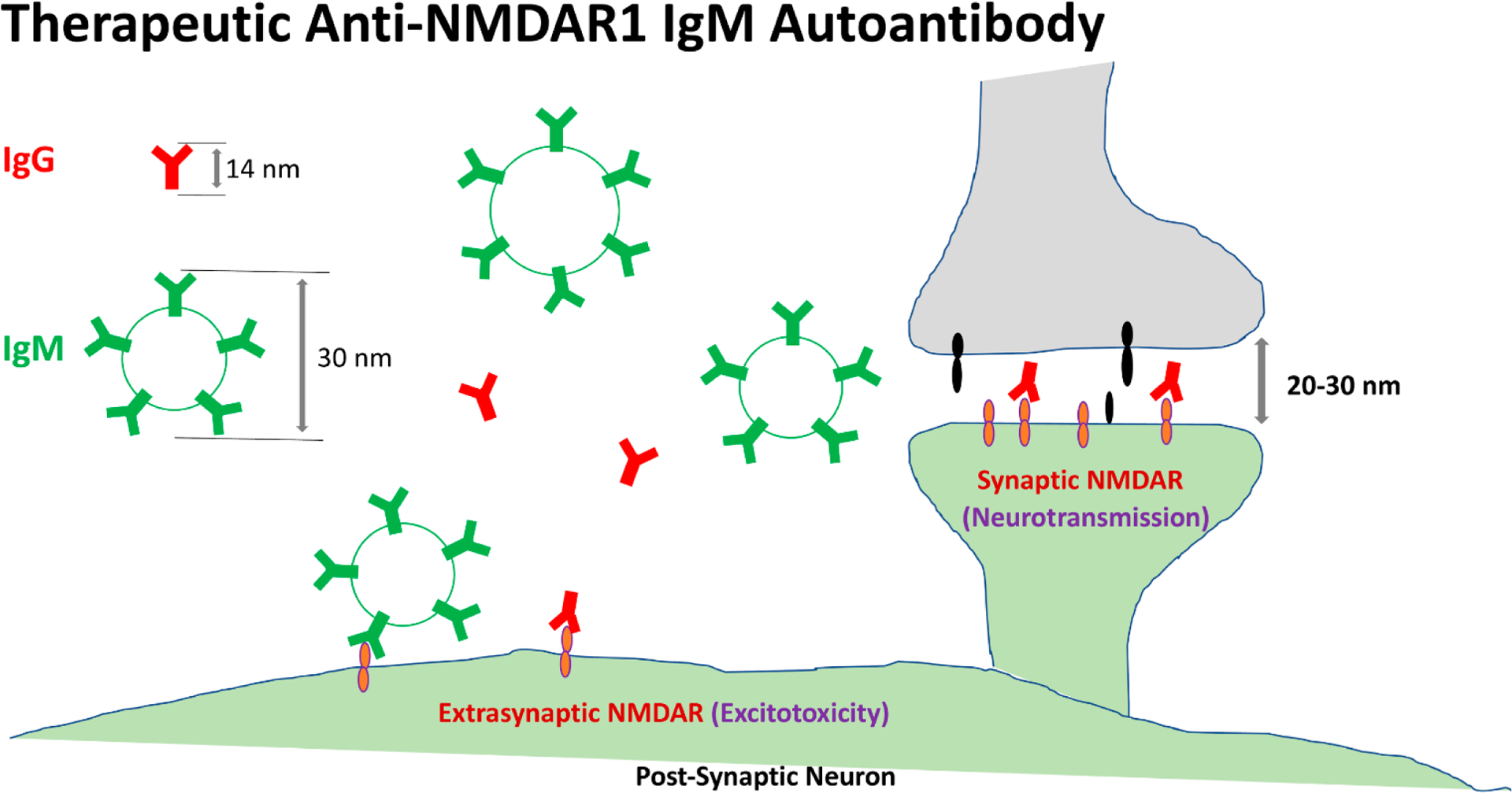
Hypothesis on two opposing effects of IgM and IgG anti-NMDAR1 autoantibodies. Synaptic NMDARs within synaptic cleft (width: 20–30 nm) can be accessed by IgG anti-NMDAR1 autoantibodies, but not IgM anti-NMDAR1 autoantibodies (pentamer diameter: 30 nm) due to their large physical size. Binding of IgM anti-NMDAR1 autoantibodies to extrasynaptic NMDARs suppresses neuronal excitotoxicity and thereby pro-cognitive.

Anti-NMDAR1 autoantibodies in blood were reported to provide protections against neuronal excitotoxicity caused by excessively released glutamate in stroke and epilepsy [28], but were also reported to impair cognitive function [2]. We propose that different isotypes of anti-NMDAR1 autoantibodies offer a plausible explanation for their beneficial and detrimental effects. According to antibody structure (Figure 1), IgG is a monomeric antibody with a planar structure (14.5 nm × 8.5 nm × 4 nm) [29]. Similar to IgG, IgA can be either monomeric or dimeric (~16.9 nm) [30]. However, IgM is a pentameric antibody that is a nonplanar, mushroom-shaped mega-molecule with a diameter of ~30 nm [31,32]. IgM hexameric antibody is even larger with a diameter of ~40 nm. In the mammalian central nervous system, the width of synaptic cleft between the axon terminal of the presynaptic neuron and the membrane of the postsynaptic neuron is ~20–30 nm [33]. Since the synaptic cleft is not an empty space but partially filled with many different synaptic proteins, size limit for molecule diffusion into the cleft is likely even smaller. Conceivably, anti-NMDAR1 IgM autoantibodies are physically too large to enter the synaptic cleft to bind NMDARs and therefore cannot inhibit synaptic NMDAR-mediated neurotransmission in both modulation of cognitive functions and pro-survival signaling. However, no such physical restriction exists for IgM anti-NMDAR1 autoantibodies to bind extrasynaptic NMDARs. Inhibition of extrasynaptic NMDARs by IgM anti-NMDAR1 autoantibodies would suppress neuronal excitotoxicity to provide neuroprotection and contribute to pro-cognitive functions. In contrast to large IgM anti-NMDAR1 autoantibodies, small IgG and IgA anti-NMDAR1 autoantibodies can enter the synaptic cleft to bind synaptic NMDARs to impair cognitive functions and suppress neuronal pro-survival signaling, albeit binding extrasynaptic NMDARs to suppress neuronal excitotoxicity. The beneficial effects of IgG and IgA anti-NMDAR1 autoantibodies on neuroprotection may be overridden by their harmful effects on cognition and neuronal survival. Therefore, blood circulating anti-NMDAR1 IgM autoantibodies could be both pro-cognitive and neuroprotective, whereas blood circulating anti-NMDAR1 IgG and IgA autoantibodies are detrimental to cognitive function. Experimental evidence of IgM exclusion from the synaptic cleft needs to be demonstrated, given that antibody shapes may be flexible. For size exclusion, the larger IgM hexamers may be a better choice than IgM pentamers for development of therapeutic anti-NMDAR1 autoantibodies.

## LONG-LASTING PREVENTIVE AND THERAPEUTIC AUTOANTIBODIES

Suppressing glutamate excitotoxicity has been the focus of drug discovery for neurological diseases, neurodegenerative diseases, and psychiatric disorders. Current small molecule NMDAR anatgonists including memantine lack sufficient specificity to target extrasynaptic NMDARs for suppression of excitotoxicity without also inhibiting synaptic NMDARs that are pro-survival and essential for cognition. Psychotomimetic side effects are common for these small molecules. In contrast, IgM anti-NMDAR1 autoantibodies cannot access synaptic NMDARs but specifically inhibit extrasynaptic NMDARs to suppress glutamate excitotoxicity, indicating a superior specificity. Furthermore, IgM anti-NMDAR1 autoantibodies may be pro-cognitive in healthy persons, since suppression of extrasynaptic NMDARs by low doses of memantine improves cognition in both humans and animals. At last, in contrast to small molecule NMDAR anatgonists that have a half-life of a few hours, IgM antibodies have a half-life of ~ 5–8 days in human blood. Therefore, IgM anti-NMDAR1 autoantibodies may provide a feasible early prevention against excitotoxicity from stroke or traumatic brain injury where the BBB is disrupted, long-lasting neuroprotection in Alzheimer’s disease where the BBB may be compromised, and cognitive enhancement in schizophrenia and other psychiatric disorders where a baseline level of the IgM autoantibodies crossovering the BBB may be beneficial.

## Data Availability

Data sharing not applicable to this article as no datasets were generated or analyzed during the current study.
